# Are Physicians Ready to Prevent Osteoporotic Fractures in Hemodialysis Patients?

**DOI:** 10.5812/ijem.6239

**Published:** 2012-09-30

**Authors:** Antonino Lasco, Antonino Catalano, Nancy Morabito

**Affiliations:** 1 Department of Internal Medicine, University of Messina, Messina, Italy

**Keywords:** Osteoporotic Fractures, Hemodialysis Patients

Dear Editor,

Chronic kidney disease (CKD) is associated with many adverse outcomes including mineral and bone disorders. The prevalence of fractures among patients with CKD exceeds that of the general population, due to impairments in bone quantity, bone quality, and abnormalities of neuromuscular function with profound effects on morbidity and mortality (1). Type 2 diabetes mellitus (T2DM), that is a major cause of CKD, appears per se associated with an increased fracture risk despite a higher bone mineral density (BMD). The heavy burden of fractures in T2DM is much more due to poor bone quality than BMD, and bone quality is not detected by common diagnostic tools such as dual energy X-ray absorptiometry (2).

Little is known about the efficacy of antiosteoporotic drugs in subjects undergoing hemodialysis, as these patients are often excluded in large randomized controlled trials.

Raloxifene, a selective estrogen receptor modulator, prevents bone loss in postmenopausal women and reduces risk of vertebral fracture (3). Although the effects on bone density and biochemical markers were about half those observed in women treated with alendronate, the reduction in vertebral fracture risk was similar to alendronate, suggesting that other factors, beyond BMD, contribute to prevention of fractures.

Saito et al. have recently reported the effects of one year raloxifene treatment on bone metabolism in hemodialysis women with and without T2DM (4). Data from Saito et al. evidence a significant increase of bone density evaluated by calcaneal quantitative ultrasound and a slight not significant reduction of the bone resorption marker NTx, both in T2DM and non T2DM women treated with raloxifene.

Raloxifene is metabolized in the liver, and the resulting glucuronides are excreted in the bile, thus CKD is expected not to affect the pharmacokinetics of raloxifene. Hemodialysis patients with T2DM usually show low bone turnover rate, therefore raloxifene, that is only a mild bone resorption inhibitor seems a valid choice. Raloxifene, differently from hormone replacement therapy (HRT), reduces also the incidence of breast cancer, but, as to HRT, increases the incidence of venous thromboembolic events, thus caution is advised for the treatment of patients with high cardiovascular risk such as in CKD. Moreover, data on the possible effects of raloxifene on the insulin-glucose homeostasis are few, but raloxifene does not significantly affect glycemic control (5).

The longer life expectancy obtained through hemodialytic programs in patients with CKD, should encourage researchers to look forward to bone health and reduction of the fracture risk.

Unfortunately, to date, there are limited data on fracture risk assessment and fracture preventions with antiosteoporotic drugs in the hemodialytic population. What has been shown by Saito et al. on surrogate markers of bone strength (BMD and bone turnover) is encouraging for further prospective intervention studies to verify the long term safety and antifracture efficacy of raloxifene in T2DM hemodialysis patients.
